# Influence of the way of reporting alpha-Amylase values in saliva in different naturalistic situations: A pilot study

**DOI:** 10.1371/journal.pone.0180100

**Published:** 2017-06-27

**Authors:** María Dolores Contreras-Aguilar, Damián Escribano, Silvia Martínez-Subiela, Silvia Martínez-Miró, Mónica Rubio, Asta Tvarijonaviciute, Fernando Tecles, Jose J. Cerón

**Affiliations:** 1 Clinic Analysis Interdisciplinary Laboratory (Interlab-UMU), Campus of Excellence Mare Nostrum, Murcia, Spain; 2 Departament of Animal Production, Veterinary school, Campus of Excellence Mare Nostrum, Murcia Spain; 3 Department of Medicine and Animal Surgey, Veterinary school, Universitat CEU Cardenal Herrera, Valencia, Spain; Radboud Universiteit, NETHERLANDS

## Abstract

The objective of this pilot study was to compare the different ways of measuring salivary alpha-amylase (sAA, enzymatic vs. concentration) and to evaluate the influence that the different ways of reporting the results can have in sAA interpretation. For this purpose, sAA was measured by direct quantification and also by an enzymatic assay in three different naturalistic situations, a physical stressor (situation 1) and two mental stressors of different intensity (situations 2 and 3). The results were expressed in three different ways (without correction, multiplied by flow rate and divided by protein concentration). sAA concentration and activity increased just after situations 1 and 3. When values were multiplied by the flow rate, significant changes after situation 1 were detected only for sAA activity but not for sAA concentration, being these changes of lower significance and magnitude that those observed for sAA activity without any correction. In addition, a significant increase in sAA activity was found at T+15 in situation 2. In situation 3 the significant decrease in sAA at T+15 disappeared. When values were divided by protein concentration, there were no significant changes in situations 1 or 3, but a decrease in situation 2 at T+0 and an increase at T+15. sAA activity and concentration showed a significant correlation in all situations. This pilot study points out that the way of expressing sAA can influence the results obtained in different stress models and also their interpretation. Therefore, how sAA is reported and the factors involved in the different ways of expressing sAA, should be taken into consideration for an objective interpretation of sAA values.

## Introduction

Salivary alpha-amylase (sAA; EC 3.2.1.1) is secreted by the parotid gland in response to adrenergic activity and increases in psychological and intense physical stress situations [1–3]. Thus, sAA has been suggested as a marker of sympathetic activation having the advantages of using saliva as a sample, which is easy to obtain and being non-invasive. It has been widely used in recent studies as a tool to evaluate stress [1,4,5].

Usually sAA is measured in saliva by its enzymatic activity using spectrophotometric assays. These are reported as international units of sAA activity per millilitre of sample analysed (IU/mL) [6]. However, some authors recommended measuring the amylase output, which also takes into consideration the saliva flow rate. This in defined as millilitres of saliva produced per minute and, therefore, they report sAA values as international units of sAA activity produced by an individual in one minute (IU/min) [4,7–9]. In addition, values normalized by protein content in saliva have been previously used to report other analytes such as IgA [10,11].

The direct measurement of the concentration of sAA has been reported as an alternative to measure the sAA activity, because direct quantification would not be influenced by factors such as enzymatic-hydrolysis of substrate or saliva pH, which can produce variations in the enzymatic assays [6]. sAA concentration has been shown to be more sensitive than the enzymatic activity in detecting stress in the horses’ [12]. However current research suggests few studies have assessed the dynamics of sAA concentration in humans in situations of stress [13].

The objective of this study was to compare the different methods of measuring sAA (enzymatic vs. concentration) and to evaluate the influence the different ways of reporting the results can have in sAA interpretation. For this purpose, sAA was measured in three different naturalistic situations by an enzymatic assay and also by direct quantification. The results were expressed in three different ways: without correction, multiplied by flow rate and divided by protein concentration. The naturalistic situations in which sAA were measured were an indoor soccer match (situation 1), which have been proven to produce an increase in sAA activity due to physical stress [14], and two different academic activities: situation 2 involved the resolution of clinical cases which are considered to induce low stress, and the second one consisted of collecting blood from pigs (situation 3), which is regarded as inducing high stress to students.

## Materials and methods

### Participants and description of stress situations

Veterinary undergraduates from University of Murcia participated in this experiment. All of them were informed about the procedure, sampling methods and the objective of the experiment. All participants signed a consent form. The Murcia University Ethics Committee (Spain) approved this study.

The final football match of an indoor, internal Veterinary School league was used as situation 1. This occurred in May 2016. Saliva samples were taken from 13 males students (mean age = 22±1.5 years) five minutes just before the match (T-0, 20:00) and five minutes after the match (T+0, 21:00).

Situation 2 was an academic activity of apparently low stress. This consisted of the resolution of clinical cases by second year students in the *Pathophysiology* course. It was considered a low stress activity because students did not have pressure to solve the cases and it did not count toward the final examination grade of the course. Saliva samples of 17 female student volunteers (average age = 19.38 ±0.62 years) were collected just before the beginning of the practice (T-0, 12:00), just after ending it (T+0, 13:30) and fifteen minutes later (T+15, 13:45).

Situation 3 was an academic activity of apparently high stress. This entailed collecting pig blood from Farm Veterinary Teaching at the University of Murcia. It was undertaken by 16 female students (average age = 24.13±3.03 years) enrolled on the *Clinical Rotations* fifth year course. Samples were collected from 11:30 a.m. to 14:00 a.m: at least fifteen minutes before blood collection (T-0), immediately after blood collection (T+0) and fifteen minutes later (T+15). The blood collection took 19,33±20.81 seconds of average, and the mean number of tries was 1,75±0.93 times.

### Subject exertion and stress perception

To quantify the intensity of the final indoor football match, the rating of perceived exertion (RPE) was recorded using the Borg´s CR-10 scale [14,15] where intensity perception is represented by ‘*nothing at all*’, ‘*extremely light*’, ‘*very light*’, ‘*light*’, ‘*somewhat hard*’, ‘*hard*’, ‘*very hard*’, ‘*extremely hard*’ and ‘*maximum exercise*’ and evaluated using a 10-point scale.

The subject stress perception of academic practices was estimated using the Academic Stress Inventory questionnaire [16,17], where the stress is evaluated on cognitive, motor and physiologic levels that are combined and interpreted on a single scale. Participants rated how much they agreed with 11 different items using a 5-point graded response scale ranging from ‘*not at all’* (1) to ‘*very much’* (5). The scores from these items were combined, and a mean score was calculated for each participant. Cronbach´s alpha coefficient was used to evaluate the questionnaire [18–20].

### Sampling

Saliva was collected for one minute by passive flow [6,21] under supervision, using 5 mL standard microcentrifuge polystyrene tubes with round bottoms (12 X 75 mm) [6]. To minimise any potential physiological effects about responses to sAA, one hour before the beginning of the saliva collection, participants were not allowed to eat, have coffee or caffeinated soft drinks, or consume dairy products; alcohol consumers and smokers were excluded from the test [6]. Participants rinsed their mouth with clear water to avoid contamination of saliva samples with food components. Each sample was refrigerated or stored on ice until arrival at the laboratory. Firstly, samples were weighed and then they were centrifuged at 4.500 x g for 10 min at 4°C to remove cells [21]. The supernatant was transferred in 1.5 mL eppendorf tubes and stored at -80°C until analysis.

### Enzymatic assay

sAA activity was measured using a colorimetric commercial kit (Alpha-Amylase, Beckman Coulter Inc., Fullerton, CA, USA) following the International Medicine (IFCC) method [6,22], as previously reported and validated [16].

### Time-resolved immunofluorometric assay (TR-IFMA)

The assay was as a noncompetitive indirect sandwich method based on anti-human-α-amylase polyclonal antibody biotin-labeled as a capture reagent and the anti-human-α-amylase polyclonal antibody Eu3+-chelates labeled as a detector. Streptavidin-coated plates (Streptavidin Microtitration Strips, DELFIA, PerkinElmer, Turku, Finland) were used for the development of this assay.

The fluorometric assay was validated in the researcher’s laboratory, using three salivary pools with a low, medium and high sAA mean concentration (123, 329 and 1722 μg/mL, respectively) for the determination of intra and inter-assay precision. For the intra-assay precision calculation, each sample was measured five times in a single run, whereas for the inter-assay precision each sample was measured on five different days. The coefficients of variation (CV) were 5.12%, 1.91% and 13.83% for intra-assay; and 13.95%, 13.74% and 13.69% for inter-assay, respectively. Linearity under dilution test in the two pools used yielded Pearson correlation coefficients of r = 0.986 and r = 0.998. The limit of detection and the lower limit of quantification calculated were 1.98 x 10^−4^ μg/mL and 41.42 μg/mL, respectively.

### Flow rate

The salivary flow rate was obtained by dividing the volume of the saliva collected by the time of the sampling period (1 min) [6]. The volume of the saliva collected was obtained by subtracting the empty tube weight from the saliva-filled one, and the grams obtained were considered equivalent to millilitres [23]. Both the activity and the sAA amount were later multiplied by flow rate (IU/min and μg/min, respectively).

### Total protein quantification

A colorimetric kit to measure urine and Low-Complexity Region (LCR) proteins (Protein in urine and CSF, Spinreact, Spain) was used to measure the content of total proteins (Prot. T) on saliva expressed in mg/mL. The method was adapted to an automatic analyzer (Olympus UA600, Olympus Diagnostica GmbH, Ennis, Ireland) following the manufacturer’s guidelines. Results from sAA activity and the direct quantification in the three evaluated situations were divided by its content of protein (IU/mg and μg/mg, respectively).

### Statistical analysis

Arithmetic means, medians, coefficients of variation and linear regression analyses were calculated using routine descriptive statistical procedures by spreadsheet (Excel 2000, Microsoft Corporation, Redmond, Washington, USA). Data were assessed for normality by using the Shapiro-Wilk test, they showed a non-parametric distribution and were transformed logarithmically by applying the formula ln x = ln(x + 1) before the statistical analyses [6,24] that restored normality. Paired Student’s t-test (2-tailed) in situation 1 and one-way ANOVA of repeated measures and Fisher’s LSD test in situation 2 and 3 were used to determine if values evaluated at different times in each group were statistically different. A stand-alone power programme for statistical testing commonly used in social and behavior research, (G-Power) [25] was employed to compute achieved power (1-β error probability) in each naturalistic situation, with a significance level of α = 5%. The Spearman correlation test was used to compare both measurement methods. The significance level used in each case was *P*<0.05. These statistical analyses were calculated using the Graph Pad Prism 6 (GraphPad Software, San Diego, CA, USA).

## Results

### Subject exertion and stress perception

The median RPE score for situation 1 was 7, indicating that the exertion was between ‘*hard’* and ‘*extremely hard’*.

Mean scores in the questionnaire made after the academic situations were 1.43±0.49 for situation 2 and 1.53±0.43 for situation 3. Cronbach’s alpha coefficients were 0.83 and 0.91 respectively for each group.

### Influences of stress situations in the flow rate and the total protein

**Fig 1:**
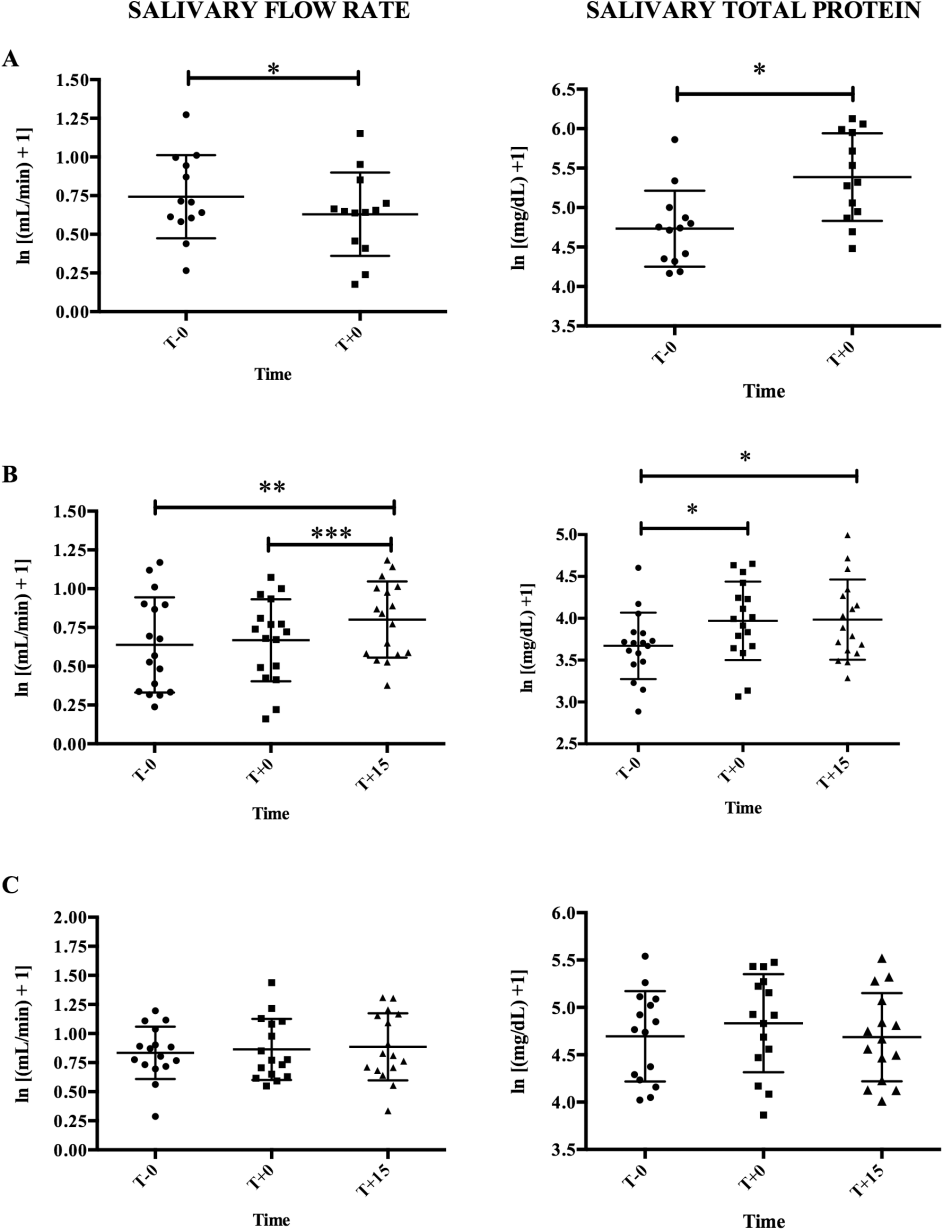
Salivary flow rate (left) and salivary total protein (right) in the three situations expressed as log transformed (ln x = ln(x + 1)). (A) A final football indoor match (situation 1), five minutes before match (T-0) and five minutes after match (T+0); (B) an academic activity consisted of the resolution of clinical cases (situation 2), just before (T-0), just after (T+0) and fifteen minutes after (T+15) practice; (C) an activity collecting pig’s blood (situation 3), at least fifteen minutes before (T-0), just after (T+0) and fifteen minutes after (T+15) the collection. Graphs show means ± Standard deviation (SD). Asterisks indicate significant post-hoc difference (Fisher LSD test): * *P*<0.05; ** *P*<0.01; *** *P*<0.001.

Salivary flow and total protein are showed in Fig 1. Salivary flow after situation 1 was significantly lower (0.85-fold, *t*_12_ = 2.29, P<0.05, *dz* = -0.63) than before. Significant differences were observed in situation 2 (*F*_1.57,25.20_ = 7.53, P<0.01, *f* = 0.26), with an increase fifteen minutes after (T+15) compared with just before (T-0) and just after (T+0). This increase was of 1.26-fold when compared to T-0 and 1.20-fold when compared to T+0. Salivary total protein after situation 1 was significantly higher (1.14-fold, *t*_12_ = 2.95, P<0.05, *dz* = 0.82) than before, and yielded significant differences between times in situation 2 (*F*_1.73,27.64_ = 6.09, P<0.01, *f* = 0.32), with an increase at T+0 (1.08-fold) and at T+15 (1.09-fold) compared to T-0. No significant changes in flow rate (*F*_1.98,29.72_ = 0.48, P = 0.62, *f* = 0.08) and salivary total protein (*F*_1.65,23.13_ = 1.01, P = 0.37, *f* = 0.14) were observed in the situation 3.

### sAA in stress situations

**Fig 2:**
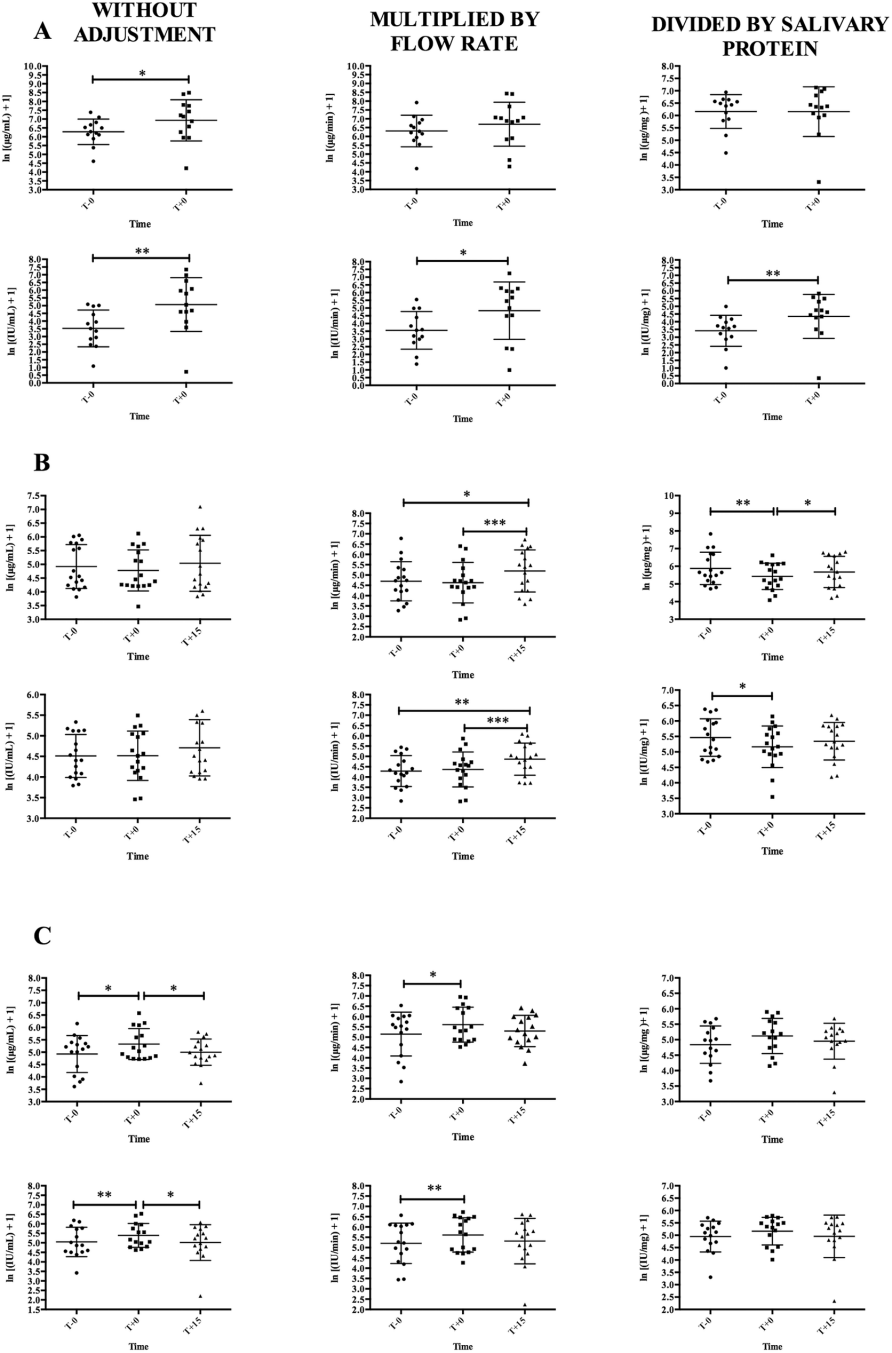
Different ways to express salivary alpha-amylase (sAA) concentration (upper graph) and activity (lower graph) in three biological situations expressed as log transformed (ln x = ln(x + 1)). (A) A final football indoor match (situation 1), five minutes before match (T-0) and five minutes after match (T+0); (B) an academic activity consisting of the resolution of clinical cases (situation 2), just before practice (T-0), just after (T+0) and fifteen minutes after (T+15) practice; (C) an activity collecting pig’s blood (situation 3), at least fifteen minutes before (T-0), just after (T+0) and fifteen minutes after (T+15) the collection. Graphs show means ± Standard deviation. Asterisks indicate significant post-hoc difference (Fisher LSD test): * *P*<0.05; ** *P*<0.01; *** *P*<0.001.

sAA values in the different stress situations are shown in Fig 2. sAA without any type of adjustment was significantly higher after situation 1 than before when measured by sAA concentration (1.10-fold, *t*_12_ = 2.25, P<0.05, *dz* = 0.62) and also by enzymatic activity (1.49-fold, *t*_12_ = 3.86, P<0.01, *dz* = 1.07). No significant changes were presented in situation 2 in sAA concentration (*F*_1.47,23.53_ = 1.41, P = 0.26, *f* = 0.12) or sAA activity (*F*_1.631,26.10_ = 1.84, P = 0.18, *f* = 0.15). In situation 3, sAA showed significant changes in concentration (*F*_1.79,26.81_ = 4.60, P<0.05, *f* = 0.28) and activity (*F*_1.61,22.56_ = 4.25, P<0.05, *f* = 0.22), with an increase just after (T+0) blood collection (1.08-fold for concentration and 1.07-fold for activity) and a decrease fifteen minutes after (0.94-fold and 0.93-fold, respectively).

When sAA results were multiplied by the flow rate, higher sAA enzymatic activity after situation 1 than before was observed, although this change has a lower magnitude and significance level (1.36-fold, *t*_12_ = 3.01, P<0.05, *dz* = 0.85) compared with sAA expressed without any adjustment. No significant changes were observed between times in the sAA concentration corrected by flow (*t*_12_ = 1.26, P = 0.23, *dz* = 0.35) in situation 1. In situation 2, sAA concentration (*F*_1.51,24.18_ = 4.88, P<0.05, *f* = 0.26) and enzymatic activity (*F*_1.63,26.16_ = 7.09, P<0.01, *f* = 0.32) significantly changed showing an increase at T+15 compared to T-0 (1.10-fold for concentration and 1.14-fold for activity) and T+0 (1.12-fold for concentration and 1.11-fold for activity). In situation 3, when sAA results were multiplied by flow rate, significant changes between times were observed in sAA concentration (*F*_1.91,28.71_ = 4.41, P<0.05, *f* = 0.21) and activity (*F*_1.63,26.10_ = 3.25, P<0.05, *f* = 0.20), with an increase at T+0 (1.09-fold and 1.08-fold, respectively) compared to T-0.

When sAA was normalized by its protein amount, there were no significant changes in sAA concentration (*t*_12_ = 0.03, P = 0.97, *dz* = -0.01) after situation 1, and in sAA concentration (*F*_1.94,27.13_ = 2.50, P = 0.1, *f* = 0.20) and activity (*F*_1.81,25.41_ = 1.70, P = 0.20, *f* = 0.15) in the situation 3. In the situation 2, sAA concentration (*F*_1.49,23.86_ = 5.56, P<0.05, *f* = 0.22) and activity (*F*_1.63,26.10_ = 3.25, P<0.05, *f* = 0.20) showed significant changes; with a decrease at T+0 compared to T-0 for sAA concentration (0.92-fold) and activity (0.94-fold), and a significant increase at T+15 compared to T+0 for sAA concentration (1.04-fold).

A Statistical power higher than 0.80 was only achieved in situation 1 with sAA results expressed as IU/mL, IU/min and IU/mg; and in situation 2 with sAA results expressed as IU/min.

**Fig 3:**
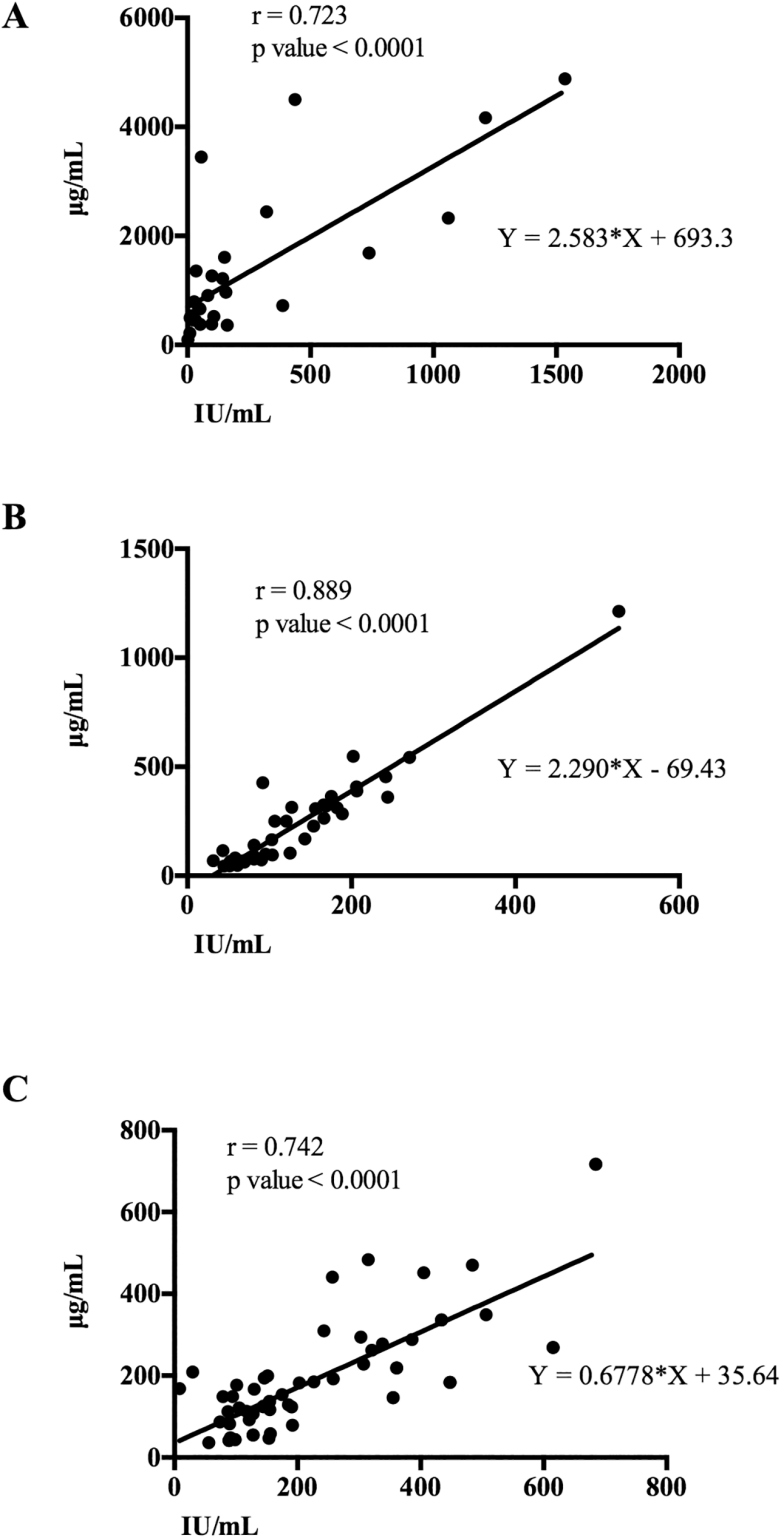
Linear regression of correlation plots. A correlation study was made to compare sAA enzymatic activity with quantification by fluorometry in three situations consisting of an indoor football match (A) or situation 1, a clinical cases practice (B) or situation 2 and a blood collecting activity (C) or situation 3. r = Spearman r-value.

sAA values measured by activity (IU/mL) and concentration (μg/mL) showed a significant correlation (p<0.0001) with Spearman r-values of 0.723, 0.889 and 0.742 (Fig 3) for situation 1, situation 2 and situation 3, respectively.

## Discussion

The ways to express human sAA are ambiguous and they make the bulk of sAA findings difficult to interpret, creating confusion between sAA activity, sAA concentration and sAA secretion [4]. This pilot study evaluated how the way to express sAA can influence the values reported in different situations of stress.

In the example of situation 1, an indoor football match, high intensity with a median PRE in the Borg scale of 7 was observed. The higher levels found in sAA enzymatic activity and concentration without any correction after situation 1 corroborated previous reports and would confirm that physical exercise produces increases in sAA [3,14]. Although there are previous reports published in which it is assumed that a significant increase of sAA in a physical stress using two times (before and after the stress) is as a result of a stress response [26–29], ideally studies with at least three measurement points (baseline, peak, recovery) should be performed to confirm the sAA increases. In addition, our results of sAA enzymatic activity in the two academic situations confirmed expectations. There were no changes in sAA in the clinical cases practice (situation 2) as there was no pressure for the students because the activity of solving clinical cases did not count toward the final examination grade of the course. Whereas in the blood collecting practice (situation 3) there was an increase in sAA that would reflect a stress for the students that may be due to their lack of experience in taking pig’s blood samples, and/or the impact on the students of the reaction of the pigs reactions to nose snaring. These situations can cause stress especially in students more sensitive to animal welfare issues. In the situation 3, the questionnaire indicated a low degree of stress in students and therefore did not agree with sAA values. This lack of agreement between questionnaires and salivary stress biomarkers such as cortisol, has also been identified in previous reports about veterinary students responses to different activities, for example public oral speech [16], gynaecological examination of horses [30], and in others psychological studies [31]. In addition, Campbell and Ehlert [31] have identified various elements that potentially contribute to the apparent dissociation between the emotional and the physiological response in acute psychosocial stress models such as underlying psychological traits and states.

When multiplied by the salivary flow rate, no changes in sAA concentration were detected in situation 1, and the changes in sAA activity were of lower magnitude and significance than without correction. This change in the football match could be due to the fact that the salivary flow rate was lower after the match and therefore if multiplied by sAA, it will produce an overall reduction in the value. Since saliva flow is related mainly with parasympathetic activity [32], it can be stated that when sAA is expressed by flow, both a sympathetic (measured by sAA) and a parasympathetic component (measured by flow) are evaluated at the same time and this can produce contradictory results. Therefore it could be recommended that in these situations both components, sAA and salivary flow, should be measured and evaluated separately instead of being combined, as happens when sAA multiplied by flow is reported. However, in the situation 2, sAA increases at T+15 when multiplied by salivary flow rate. It could be postulated that this academic situation could lead to an activation of the parasympathetic system and therefore an increase in flow, and when it was multiplied by sAA would produce an overall increase in the value. If this was the case, the multiplication by salivary flow rate could identify situations in which there is only a parasympathetic activation.

When sAA was corrected by salivary protein, the results compared to values without correction changed in all the experimental procedures. There was a disappearance of the significance of the sAA increases in activities of situation 1 and 3, and a decrease in sAA after the situation 2 was observed. It could be proposed that since the saliva protein is a very heterogeneous mix of different compounds, some of its compounds, in addition to sAA, could increase or decrease differently in the different stress situations and therefore salivary total protein concentration excreted in saliva cannot be considered as a constant factor. Walsh et al. [33] indicated that if protein concentration on saliva is used in a similar way to creatinine concentration on urine to correct differences in flow because its excreted concentration is relatively constant, the argument collapses when changes in protein secretion in saliva are observed in different stress activities. The increase in sAA concentration at T+15 that appears when corrected by total protein seems too late compared with previous reports that indicated that increases in sAA occur not later than 10 min [34]. This result could be related with the variability in the response of total protein in saliva, as previously indicated. Although further studies should be made to clarify this, it could be suggested to take special consideration interpreting the sAA values when corrected by saliva protein.

The correlation between sAA activity and concentration not corrected in the three situations was higher than 0.700, showing a high significance. This relationship is higher than data from Mandel et al. [35], who found correlations of 0.61. Overall sAA activity and concentration have a positive correlation and showed variations with a similar tendency in the different stress models. sAA concentrations showed a lower inter-individual variability than sAA activities, as can be visually observed from the lower standard deviations that appear in the figures. However, in general the changes in concentration were of lower significance than those produced in the enzymatic activity, indicating that sAA enzymatic activity was more sensitive than concentration to detect those changes induced by stress in the situations of the study. A possible reason for these differences could be that the TR-IFMA assay could measure specific isoforms of sAA that could differ in their enzymatic activities [36]. Further research should be conducted to test this hypothesis. In addition, further research should be undertaken to evaluate if sAA concentration can be more appropriate to detect changes than sAA activity in other stress models such as has been reported for horses [12].

The statistical power was not higher than 0.80 in all the different ways to express sAA in the biological situations studied in our report. In addition, the diverse sample population and measurement time points used in each situation can represent a limitation of the study. Therefore, overall this report should be considered as a pilot one and further studies involving a larger population and also using the same individuals under different stressful situations, should be performed in the future to confirm our findings.

## Conclusions

The use of sAA values expressed taking in to account salivary flow or protein concentration can produce variability in the results of sAA in different stress models, compared with the results expressed without any correction. These variations can be due to many factors, such as changes in saliva flow rate or the increase or decrease in other salivary proteins at different stress situations. This pilot study points out that the way of expressing sAA can influence the results obtained in different stress models and also their interpretation. Therefore how sAA is reported and the factors involved in the different ways of expressing sAA, should be taken into consideration for an objective interpretation of sAA values.
